# 2-[1,1-Dioxo-2-(2,4,5-trifluoro­benz­yl)-2*H*-1,2-benzothia­zin-4-yl]acetic acid

**DOI:** 10.1107/S1600536812015942

**Published:** 2012-04-13

**Authors:** Yanchun Yang, Youzhu Yu, Changjin Zhu

**Affiliations:** a Department of Applied Chemistry, Beijing Institute of Technology, Zhongguancun South Street, 100081 Beijing, People’s Republic of China; b Department of Chemistry and Environmental Engineering, Anyang Institute of Technology, Henan 455000, People’s Republic of China

## Abstract

In the title compound, C_17_H_12_F_3_NO_4_S, the heterocyclic thia­zine ring adopts a half-chair conformation with the S and the N atoms displaced by −0.608 (3) and 0.105 (3) Å, respectively, from the mean plane formed by the remaining ring atoms. The dihedral angle between the two benzene rings is 36.63 (8)° and the acetic acid group is inclined at right angles [89.78 (8) °] to the mean plane formed by the C atoms of the thia­zine ring. The crystal structure features O—H⋯O and C—H⋯O hydrogen bonds.

## Related literature

For pharmaceuticals properties of benzothia­zines, see: Zia-ur-Rehman *et al.* (2006 ▶). For synthetic details of the title compound, see: Chen *et al.* (2011 ▶). For related structures, see: Ahmad *et al.* (2008 ▶); Zia-ur-Rehman *et al.* (2008 ▶). 
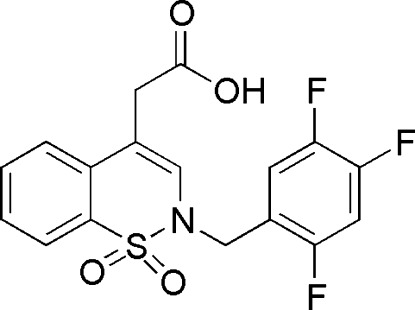


## Experimental

### 

#### Crystal data

C_17_H_12_F_3_NO_4_S
*M* *_r_* = 383.34
Monoclinic, 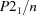

*a* = 8.3290 (17) Å
*b* = 23.141 (5) Å
*c* = 8.6692 (17) Å
β = 90.93 (3)°
*V* = 1670.7 (6) Å^3^
*Z* = 4
Mo *K*α radiation
μ = 0.25 mm^−1^
*T* = 293 K
0.20 × 0.20 × 0.20 mm

#### Data collection

Bruker APEXII CCD diffractometer
22476 measured reflections
4158 independent reflections
3646 reflections with *I* > 2σ(*I*)
*R*_int_ = 0.023

#### Refinement

*R*[*F* ^2^ > 2σ(*F* ^2^)] = 0.039
*wR*(*F* ^2^) = 0.108
*S* = 1.03
4158 reflections
239 parameters
H atoms treated by a mixture of independent and constrained refinement
Δρ_max_ = 0.32 e Å^−3^
Δρ_min_ = −0.40 e Å^−3^

### 

Data collection: *APEX2* (Bruker, 2005 ▶); cell refinement: *SAINT-Plus* (Bruker, 2001 ▶); data reduction: *SAINT-Plus*; program(s) used to solve structure: *SHELXS97* (Sheldrick, 2008 ▶); program(s) used to refine structure: *SHELXL97* (Sheldrick, 2008 ▶); molecular graphics: *SHELXTL* (Sheldrick, 2008 ▶); software used to prepare material for publication: *SHELXTL* and *PLATON* (Spek, 2009 ▶).

## Supplementary Material

Crystal structure: contains datablock(s) I, global. DOI: 10.1107/S1600536812015942/pv2523sup1.cif

Structure factors: contains datablock(s) I. DOI: 10.1107/S1600536812015942/pv2523Isup2.hkl

Supplementary material file. DOI: 10.1107/S1600536812015942/pv2523Isup3.cml

Additional supplementary materials:  crystallographic information; 3D view; checkCIF report

## Figures and Tables

**Table 1:** Hydrogen-bond geometry (Å, °)

*D*—H⋯*A*	*D*—H	H⋯*A*	*D*⋯*A*	*D*—H⋯*A*
O1—H1⋯O2^i^	0.84 (3)	1.83 (3)	2.675 (2)	179 (3)
C8—H8⋯O3^ii^	0.93	2.55	3.211 (2)	129
C9—H9*A*⋯O3^ii^	0.97	2.30	3.207 (2)	155
C11—H11*B*⋯O4^iii^	0.97	2.40	3.162 (2)	135

Symmetry codes: (i) 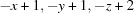; (ii) 
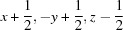
; (iii) 
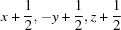
.

**Fig. 1.**
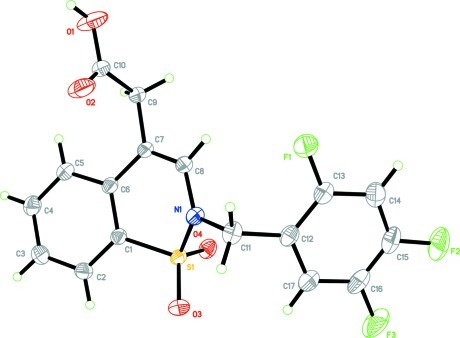
The molecular structure of the title compound with the atom numbering scheme. Displacement ellipsoids are drawn at the 30% probability level. H atoms are presented as small spheres of arbitrary radius.

**Fig. 2.**
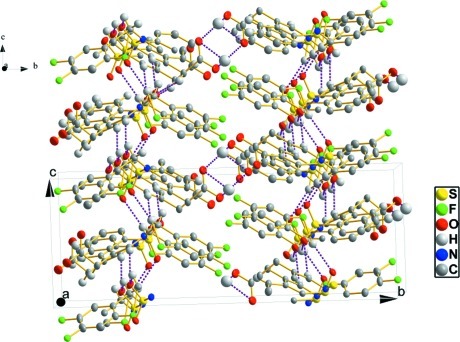
A view of the O—H···O and C—-H···O hydrogen bonds (dotted lines) in the crystal structure of the title compound.

## supplementary crystallographic information

### Comment

Benzothiazine moiety is present as the skeletal structure in several pharmaceuticals such as antibacterial, diuretic, hypoglycemic, antithyroid, and antitumor drugs (Zia-ur-Rehman *et al.*, 2006). In this article, we report the crystal structure of the title compound which has been used as aldose reductase inhibitor (Chen *et al.*, 2011).

In the title compound (Fig. 1), the heterocyclic thiazine ring adopts a half chair conformation with the S1 and the N1 atoms displaced by -0.608 (3) and 0.105 (3) Å, respectively, from the mean plane formed by the remaining ring atoms (C1/C6/C7/C8). The dihedral angle between the two benze rings (C1–C6) and (C12–C17) is 36.63 (8)° and the acetate group (O1/O2/C9/C10) is inclined at right angles (89.78 (8) °) to the mean plane formed by the C-atoms of the thiazine ring. The crystal structure is stabilized by intermolecular O—H···O and C—H···O hydrogen bonds forming a three-dimensional network (Fig. 2).

### Experimental

A mixture of methyl 2-(1,1-dioxido-2-(2,4,5-trifluorobenzyl)-2*H*-benzo[*e*][1,2] thiazin-4-yl)acetate (0.5 mmol), 1,4-dioxane (5 ml) and saturated aqueous sodium hydroxide (8 ml) was stirred at room temperature for 12 h. The alkaline suspension was adjusted to be acidic with 0.1 *M* HCl and extracted with ethyl acetate (3 *x* 30 ml). The combined organic layers were dried over MgSO_4_ and filtered. Crystals suitable for X-ray diffraction were obtained by slow evaporation of a solution of the title compound in methanol (yield = 69%).

### Refinement

H atom bonded to O1 was located from a different Fourier map and refined freely. The remaining H atoms were positioned geometrically, with C—H = 0.93 and 0.97 Å for aromatic and methylene H, respectively, and constrained to ride on their parent atoms with *U_iso_*(H) = 1.2*U_eq_*(C).

### Crystal data

**Table:**

C_17_H_12_F_3_NO_4_S	*F*(000) = 784
*M**_r_* = 383.34	*D*_x_ = 1.524 Mg m^−^^3^
Monoclinic, *P*2_1_/*n*	Mo *K*α radiation, λ = 0.71073 Å
Hall symbol: -P 2yn	Cell parameters from 9975 reflections
*a* = 8.3290 (17) Å	θ = 2.5–28.4°
*b* = 23.141 (5) Å	µ = 0.25 mm^−^^1^
*c* = 8.6692 (17) Å	*T* = 293 K
β = 90.93 (3)°	Block, colorless
*V* = 1670.7 (6) Å^3^	0.20 × 0.20 × 0.20 mm
*Z* = 4	

### Data collection

**Table:**

Bruker APEXII CCD diffractometer	3646 reflections with *I* > 2σ(*I*)
Radiation source: fine-focus sealed tube	*R*_int_ = 0.023
Graphite monochromator	θ_max_ = 28.4°, θ_min_ = 2.5°
φ and ω scans	*h* = −11→11
22476 measured reflections	*k* = −27→30
4158 independent reflections	*l* = −11→11

### Refinement

**Table:**

Refinement on *F*^2^	Primary atom site location: structure-invariant direct methods
Least-squares matrix: full	Secondary atom site location: difference Fourier map
*R*[*F*^2^ > 2σ(*F*^2^)] = 0.039	Hydrogen site location: inferred from neighbouring sites
*wR*(*F*^2^) = 0.108	H atoms treated by a mixture of independent and constrained refinement
*S* = 1.03	*w* = 1/[σ^2^(*F*_o_^2^) + (0.0536*P*)^2^ + 0.5773*P*] where *P* = (*F*_o_^2^ + 2*F*_c_^2^)/3
4158 reflections	(Δ/σ)_max_ < 0.001
239 parameters	Δρ_max_ = 0.32 e Å^−^^3^
0 restraints	Δρ_min_ = −0.40 e Å^−^^3^

### Special details

**Table:**

Geometry. All e.s.d.'s (except the e.s.d. in the dihedral angle between two l.s. planes) are estimated using the full covariance matrix. The cell e.s.d.'s are taken into account individually in the estimation of e.s.d.'s in distances, angles and torsion angles; correlations between e.s.d.'s in cell parameters are only used when they are defined by crystal symmetry. An approximate (isotropic) treatment of cell e.s.d.'s is used for estimating e.s.d.'s involving l.s. planes.
Refinement. Refinement of *F*^2^ against ALL reflections. The weighted *R*-factor *wR* and goodness of fit *S* are based on *F*^2^, conventional *R*-factors *R* are based on *F*, with *F* set to zero for negative *F*^2^. The threshold expression of *F*^2^ > σ(*F*^2^) is used only for calculating *R*-factors(gt) *etc*. and is not relevant to the choice of reflections for refinement. *R*-factors based on *F*^2^ are statistically about twice as large as those based on *F*, and *R*- factors based on ALL data will be even larger.

### Fractional atomic coordinates and isotropic or equivalent isotropic displacement parameters (Å^2^)

**Table:**

	*x*	*y*	*z*	*U*_iso_*/*U*_eq_	
S1	0.11950 (4)	0.232698 (14)	0.97865 (4)	0.03556 (11)	
F1	0.69758 (14)	0.21289 (5)	0.89004 (16)	0.0722 (3)	
F2	0.6328 (2)	0.02123 (6)	0.73067 (19)	0.0989 (5)	
F3	0.3601 (2)	0.01403 (5)	0.8862 (2)	0.0997 (5)	
O1	0.4665 (2)	0.48147 (7)	0.79699 (18)	0.0807 (5)	
H1	0.504 (4)	0.5074 (14)	0.855 (3)	0.107 (10)*	
O2	0.41807 (19)	0.43626 (6)	1.01497 (15)	0.0686 (4)	
O3	0.08188 (15)	0.19719 (5)	1.10801 (14)	0.0526 (3)	
O4	0.08941 (14)	0.20919 (5)	0.82864 (12)	0.0463 (3)	
N1	0.30918 (15)	0.25127 (5)	0.99625 (14)	0.0389 (3)	
C1	0.02950 (17)	0.30051 (6)	0.99039 (15)	0.0348 (3)	
C2	−0.11807 (18)	0.30537 (7)	1.06150 (18)	0.0419 (3)	
H2	−0.1616	0.2741	1.1136	0.050*	
C3	−0.19919 (19)	0.35725 (8)	1.0537 (2)	0.0504 (4)	
H3	−0.2977	0.3614	1.1014	0.060*	
C4	−0.1331 (2)	0.40320 (7)	0.9744 (2)	0.0525 (4)	
H4	−0.1901	0.4376	0.9658	0.063*	
C5	0.0162 (2)	0.39876 (7)	0.90794 (19)	0.0453 (3)	
H5	0.0589	0.4304	0.8568	0.054*	
C6	0.10414 (17)	0.34714 (6)	0.91652 (15)	0.0354 (3)	
C7	0.26885 (18)	0.34231 (6)	0.86404 (16)	0.0379 (3)	
C8	0.36224 (18)	0.29773 (6)	0.90869 (17)	0.0387 (3)	
H8	0.4691	0.2980	0.8792	0.046*	
C9	0.3439 (2)	0.39103 (6)	0.77500 (19)	0.0448 (3)	
H9A	0.4296	0.3755	0.7128	0.054*	
H9B	0.2636	0.4074	0.7054	0.054*	
C10	0.41079 (18)	0.43834 (6)	0.87590 (19)	0.0433 (3)	
C11	0.4227 (2)	0.21175 (7)	1.07534 (18)	0.0448 (3)	
H11A	0.3728	0.1972	1.1679	0.054*	
H11B	0.5170	0.2336	1.1071	0.054*	
C12	0.47589 (19)	0.16095 (7)	0.97972 (17)	0.0419 (3)	
C13	0.6136 (2)	0.16311 (7)	0.8936 (2)	0.0480 (4)	
C14	0.6717 (2)	0.11724 (9)	0.8094 (2)	0.0594 (4)	
H14	0.7667	0.1200	0.7549	0.071*	
C15	0.5833 (3)	0.06758 (8)	0.8099 (2)	0.0636 (5)	
C16	0.4443 (3)	0.06386 (8)	0.8907 (3)	0.0635 (5)	
C17	0.3901 (2)	0.10940 (7)	0.9768 (2)	0.0532 (4)	
H17	0.2964	0.1058	1.0329	0.064*	

### Atomic displacement parameters (Å^2^)

**Table:**

	*U*^11^	*U*^22^	*U*^33^	*U*^12^	*U*^13^	*U*^23^
S1	0.04325 (19)	0.02765 (17)	0.03590 (18)	−0.00299 (12)	0.00411 (13)	0.00177 (12)
F1	0.0606 (7)	0.0529 (6)	0.1035 (9)	−0.0118 (5)	0.0109 (6)	−0.0096 (6)
F2	0.1302 (13)	0.0558 (8)	0.1110 (11)	0.0240 (8)	0.0069 (9)	−0.0322 (7)
F3	0.1114 (11)	0.0384 (7)	0.1489 (14)	−0.0155 (7)	−0.0099 (10)	−0.0094 (7)
O1	0.1217 (14)	0.0547 (9)	0.0655 (9)	−0.0464 (9)	−0.0027 (9)	0.0112 (7)
O2	0.0991 (11)	0.0490 (8)	0.0580 (8)	−0.0326 (7)	0.0064 (7)	−0.0001 (6)
O3	0.0639 (7)	0.0421 (6)	0.0523 (6)	−0.0011 (5)	0.0144 (5)	0.0156 (5)
O4	0.0566 (6)	0.0365 (6)	0.0457 (6)	−0.0028 (5)	−0.0032 (5)	−0.0089 (4)
N1	0.0412 (6)	0.0332 (6)	0.0424 (6)	−0.0001 (5)	−0.0004 (5)	0.0018 (5)
C1	0.0411 (7)	0.0302 (6)	0.0331 (6)	−0.0014 (5)	0.0012 (5)	−0.0017 (5)
C2	0.0420 (7)	0.0416 (8)	0.0423 (7)	−0.0052 (6)	0.0048 (6)	0.0002 (6)
C3	0.0416 (8)	0.0525 (9)	0.0573 (9)	0.0042 (7)	0.0078 (7)	−0.0013 (7)
C4	0.0495 (9)	0.0419 (9)	0.0662 (10)	0.0100 (7)	0.0044 (7)	0.0025 (7)
C5	0.0517 (8)	0.0329 (7)	0.0514 (8)	0.0015 (6)	0.0044 (7)	0.0037 (6)
C6	0.0423 (7)	0.0295 (7)	0.0344 (6)	−0.0023 (5)	0.0020 (5)	−0.0015 (5)
C7	0.0459 (7)	0.0279 (6)	0.0402 (7)	−0.0049 (5)	0.0085 (6)	−0.0032 (5)
C8	0.0403 (7)	0.0327 (7)	0.0434 (7)	−0.0047 (5)	0.0063 (6)	−0.0041 (5)
C9	0.0538 (8)	0.0320 (7)	0.0491 (8)	−0.0048 (6)	0.0167 (7)	−0.0006 (6)
C10	0.0424 (7)	0.0308 (7)	0.0572 (9)	−0.0036 (6)	0.0103 (6)	0.0024 (6)
C11	0.0513 (8)	0.0433 (8)	0.0394 (7)	0.0062 (7)	−0.0103 (6)	−0.0030 (6)
C12	0.0484 (8)	0.0356 (7)	0.0413 (7)	0.0047 (6)	−0.0097 (6)	0.0019 (6)
C13	0.0512 (8)	0.0382 (8)	0.0543 (9)	0.0021 (6)	−0.0063 (7)	−0.0013 (7)
C14	0.0631 (11)	0.0552 (11)	0.0601 (11)	0.0133 (9)	0.0033 (8)	−0.0029 (8)
C15	0.0838 (13)	0.0409 (9)	0.0659 (11)	0.0163 (9)	−0.0075 (10)	−0.0110 (8)
C16	0.0782 (13)	0.0320 (8)	0.0796 (13)	−0.0022 (8)	−0.0153 (10)	−0.0013 (8)
C17	0.0579 (10)	0.0395 (8)	0.0621 (10)	0.0000 (7)	−0.0032 (8)	0.0049 (7)

### Geometric parameters (Å, º)

**Table:**

S1—O4	1.4281 (11)	C5—C6	1.403 (2)
S1—O3	1.4292 (11)	C5—H5	0.9300
S1—N1	1.6420 (13)	C6—C7	1.457 (2)
S1—C1	1.7427 (14)	C7—C8	1.345 (2)
F1—C13	1.348 (2)	C7—C9	1.5076 (19)
F2—C15	1.342 (2)	C8—H8	0.9300
F3—C16	1.350 (2)	C9—C10	1.503 (2)
O1—C10	1.2998 (19)	C9—H9A	0.9700
O1—H1	0.84 (3)	C9—H9B	0.9700
O2—C10	1.207 (2)	C11—C12	1.509 (2)
N1—C8	1.3923 (18)	C11—H11A	0.9700
N1—C11	1.4765 (19)	C11—H11B	0.9700
C1—C2	1.388 (2)	C12—C13	1.379 (2)
C1—C6	1.4049 (19)	C12—C17	1.391 (2)
C2—C3	1.379 (2)	C13—C14	1.380 (2)
C2—H2	0.9300	C14—C15	1.365 (3)
C3—C4	1.385 (2)	C14—H14	0.9300
C3—H3	0.9300	C15—C16	1.366 (3)
C4—C5	1.383 (2)	C16—C17	1.372 (3)
C4—H4	0.9300	C17—H17	0.9300
			
O4—S1—O3	117.26 (8)	C10—C9—C7	113.55 (13)
O4—S1—N1	109.80 (7)	C10—C9—H9A	108.9
O3—S1—N1	107.48 (8)	C7—C9—H9A	108.9
O4—S1—C1	109.08 (7)	C10—C9—H9B	108.9
O3—S1—C1	111.83 (7)	C7—C9—H9B	108.9
N1—S1—C1	99.97 (7)	H9A—C9—H9B	107.7
C10—O1—H1	111 (2)	O2—C10—O1	122.93 (16)
C8—N1—C11	121.67 (13)	O2—C10—C9	124.36 (14)
C8—N1—S1	117.70 (10)	O1—C10—C9	112.65 (15)
C11—N1—S1	119.28 (11)	N1—C11—C12	114.76 (12)
C2—C1—C6	122.78 (13)	N1—C11—H11A	108.6
C2—C1—S1	118.92 (11)	C12—C11—H11A	108.6
C6—C1—S1	118.10 (10)	N1—C11—H11B	108.6
C3—C2—C1	119.04 (14)	C12—C11—H11B	108.6
C3—C2—H2	120.5	H11A—C11—H11B	107.6
C1—C2—H2	120.5	C13—C12—C17	116.91 (15)
C2—C3—C4	119.63 (15)	C13—C12—C11	121.54 (14)
C2—C3—H3	120.2	C17—C12—C11	121.54 (15)
C4—C3—H3	120.2	F1—C13—C12	118.65 (15)
C5—C4—C3	121.14 (15)	F1—C13—C14	117.28 (16)
C5—C4—H4	119.4	C12—C13—C14	124.07 (16)
C3—C4—H4	119.4	C15—C14—C13	116.87 (18)
C4—C5—C6	120.89 (14)	C15—C14—H14	121.6
C4—C5—H5	119.6	C13—C14—H14	121.6
C6—C5—H5	119.6	F2—C15—C14	120.1 (2)
C5—C6—C1	116.36 (13)	F2—C15—C16	118.81 (19)
C5—C6—C7	122.84 (13)	C14—C15—C16	121.11 (17)
C1—C6—C7	120.62 (13)	F3—C16—C15	118.92 (19)
C8—C7—C6	120.81 (13)	F3—C16—C17	119.8 (2)
C8—C7—C9	118.58 (13)	C15—C16—C17	121.31 (17)
C6—C7—C9	120.18 (13)	C16—C17—C12	119.69 (18)
C7—C8—N1	124.19 (13)	C16—C17—H17	120.2
C7—C8—H8	117.9	C12—C17—H17	120.2
N1—C8—H8	117.9		

### Hydrogen-bond geometry (Å, º)

**Table:**

*D*—H···*A*	*D*—H	H···*A*	*D*···*A*	*D*—H···*A*
O1—H1···O2^i^	0.84 (3)	1.83 (3)	2.675 (2)	179 (3)
C8—H8···O3^ii^	0.93	2.55	3.211 (2)	129
C9—H9*A*···O3^ii^	0.97	2.30	3.207 (2)	155
C11—H11*A*···O3	0.97	2.47	2.877 (2)	105
C11—H11*B*···F1	0.97	2.47	2.818 (2)	101
C11—H11*B*···O4^iii^	0.97	2.40	3.162 (2)	135

Symmetry codes: (i) −*x*+1, −*y*+1, −*z*+2; (ii) *x*+1/2, −*y*+1/2, *z*−1/2; (iii) *x*+1/2, −*y*+1/2, *z*+1/2.
